# Seroepidemiology of infection with *Toxoplasma gondii* in migrant agricultural workers living in poverty in Durango, Mexico

**DOI:** 10.1186/1756-3305-6-113

**Published:** 2013-04-20

**Authors:** Cosme Alvarado-Esquivel, Federico Campillo-Ruiz, Oliver Liesenfeld

**Affiliations:** 1 Laboratorio de Investigación Biomédica, Facultad de Medicina y Nutrición, Universidad Juárez del Estado de Durango. Avenida Universidad S/N, Durango, Dgo 34000, México; 2 Servicios de Salud de Durango. Independencia y Puebla S/N, Canatlán, Durango 34450, México; 3 Institute for Microbiology and Hygiene, Campus Benjamin Franklin, Charité Medical School, Hindenburgdamm 27, Berlin D-12203, Germany; 4 Present address: Roche Molecular Diagnostics, Pleasanton, CA, USA

**Keywords:** *Toxoplasma gondii*, Seroprevalence, Migrant workers, Cross-sectional study

## Abstract

**Background:**

Migrant agricultural workers are a group of people living in poverty with poor housing, sanitary conditions and hygiene practices. Little is known about the epidemiology of infection with *Toxoplasma gondii* in migrant agricultural workers.

**Methods:**

We investigated the presence of anti-*Toxoplasma* IgG and IgM antibodies in 173 migrant workers hired for seasonal agricultural work in Durango State in northern Mexico using enzyme-linked immunoassays.

**Results:**

Of the 173 migrant workers (mean age 34.82 ± 14.01 years), 50 (28.9%) had anti-*Toxoplasma* IgG antibodies and 36 (20.8%) had anti-*Toxoplasma* IgM antibodies. Seroprevalence was not influenced by gender, age, birth place, or educational level. In contrast, seroprevalence was significantly higher in workers residing in rural areas than those in urban or suburban areas. Migrant workers suffering from memory impairment, dizziness, or syncope had significantly higher seroprevalence of anti-*T. gondii* IgG antibodies than those without such clinical features. Logistic regression analysis showed that *T. gondii* exposure was positively associated with consumption of unwashed raw vegetables (OR = 2.39; 95% CI: 1.06-5.35; *P* = 0.03) and low frequency of eating out of home (OR = 3.87; 95% CI: 1.43-10.42; *P* = 0.007), and negatively associated with national trips (OR = 0.30; 95% CI: 0.13-0.65; *P* = 0.003) and consumption of raw milk (OR = 0.40; 95% CI: 0.18-0.87; *P* = 0.02). Other behavioral characteristics including consumption of meat or untreated water were not associated with *T. gondii* infection.

**Conclusions:**

This is the first report of *T. gondii* infection in internal migrant agricultural workers living in poverty. Results deserve further investigation of causal relations between clinical symptoms and infection, and may be useful for optimal planning of preventive measures.

## Background

*Toxoplasma gondii* (*T. gondii*) is a parasite with worldwide distribution [1]. Infections with *T. gondii* may lead to serious illness affecting mostly lymph nodes, eyes, and the central nervous system [2-4]. Transmission of *T. gondii* occurs by ingesting food or water contaminated with oocysts shed by cats or by eating undercooked or raw meat containing tissue cysts [2]. There is poor knowledge about the epidemiology of *T. gondii* infection in migrant agricultural workers in the world in general [5-7], and we are not aware of any report in the medical literature about the epidemiology of *T. gondii* infection in migrant workers in Mexico in particular. It is important to study migrant agricultural workers since they live in poverty, under poor sanitation conditions and low hygiene practices. They use untreated drinking water and have poor health care services for diagnosis, treatment, and prevention of infectious diseases in general and a lack of laboratory tests for toxoplasmosis in particular. Previous studies in Durango have shown higher frequencies of *T. gondii* exposure in rural populations [8,9] than in inhabitants in the urban capital city [10-12]. Therefore, we sought to determine the seroprevalence of *T. gondii* exposure in migrant agricultural workers in Durango, Mexico and to identify their characteristics associated with *Toxoplasma* seropositivity.

## Methods

### Study design and study population

We performed a cross sectional survey from August 2010 to August 2012 in national (internal) migrant workers hired for seasonal agricultural work in Durango State, Mexico. Migrant agricultural workers in Mexico consist of a particular group of the population who move from region to region within the country to get agricultural work. Many of these itinerant agricultural workers travel from one region to another along with their family members. Local (born in the same Mexican state where they work) migrant agricultural workers outnumber those who migrate from other Mexican states or from abroad. In Durango, migrant agricultural workers come from several regions of Durango State and neighboring states from northern Mexico. The migration patterns of these workers were mostly rural-rural and few were urban–rural. During their temporary employment, migrant workers live in poor socioeconomic conditions including poor housing, food, and sanitation. Inclusion criteria for the study subjects were: 1) migrant workers involved in seasonal agricultural labor in Durango, Mexico, 2) any gender, 3) 14 years and older, 4) any socioeconomic level, and 5) that voluntarily accepted to participate. In total, 173 migrant agricultural workers were studied. They worked in the municipalities of Durango and Canatlán in the valley region of Durango State. Durango municipality has a temperate climate and Canatlán municipality has a semi-cold climate. Migrant agricultural workers had spent up to 3 months in the current work place.

### Ethical aspects

This study was approved by the Institutional Ethical Committee of the General Hospital of the Secretary of Health in Durango City. The purpose and procedures of the study were explained to all participants. A written informed consent was obtained from all participants.

### Socio-demographic, clinical, and behavioral data

We obtained the characteristics of the participants by using a standardized questionnaire. Socio-demographic data including age, gender, birth place, residence, educational level, and socioeconomic status were obtained from all participants. Clinical data explored included the presence of underlying diseases, presence or history of lymphadenopathy, frequent presence of headache, memory, reflex, hearing, and visual impairments, and a history of surgery, blood transfusion or transplants; in women, a history of abortion was also documented. Contributing and confounding risk factors of behavioral data included animal contacts, contact with cat excrement, foreign travel, type of meat consumption, frequency of meat consumption, consumption of raw or undercooked meat, unpasteurized milk, dried or cured meat, consumption of unwashed raw vegetables, fruits, or untreated water, frequency of eating out of home (in restaurants or fast food outlets), contact with soil, and types of floors at home were obtained from all participants.

### Serological examination for *T. gondii* antibodies

Serum samples were obtained from all participants and kept frozen at –20°C until analyzed. Serum samples were analyzed by qualitative and quantitative methods for anti-*T. gondii* IgG antibodies with a commercially available enzyme immunoassay “*Toxoplasma* IgG” kit (International Immuno-Diagnostics, Foster City, CA, U.S.A.). Anti-*T. gondii* IgG antibody levels were expressed as International Units (IU)/ml, and a result equal to or greater than 8 IU/ml was considered positive. In addition, sera positive for *T. gondii* IgG were further analyzed for anti-*T. gondii* IgM antibodies by a commercially available enzyme immunoassay “*Toxoplasma* IgM” kit (International Immuno-Diagnostics, Foster City, CA, U.S.A.). Such a strategy of testing anti-*T. gondii* IgM antibody only in anti-*T. gondii* IgG antibody positive individuals, and not vice versa, was selected because anti-*T. gondii* IgM antibodies disappear with time while anti-*T. gondii* IgG antibodies appear early after infection and remain over a lifetime. All tests were performed following the instructions of the manufacturer.

### Statistical analysis

The statistical analysis was performed with the aid of the software Epi Info version 3.5.3 and SPSS version 15.0. For calculation of the sample size, we used a reference [8] seroprevalence of 23.8% as expected frequency of the factor under study, 1,000 as the size of the population from which the sample was selected, a least acceptable result of 30.0%, and a confidence level of 95%. The result of the calculation was 153 subjects. We used the Pearson’s chi-square test and the Fisher exact test (when values were less than 5) for comparison of the frequencies among groups. Bivariate and multivariate analyses were used to assess the association between the characteristics of the subjects and *T. gondii* seropositivity. Variables were included in the multivariate analysis if they had a *P* value equal to or less than 0.20 in the bivariate analysis. Odds ratio (OR) and 95% confidence intervals (CI) were calculated by multivariate analysis using multiple, unconditional logistic regression. A *P* value less than 0.05 was considered statistically significant.

## Results

Of the 173 migrant workers, 50 (28.9%) had anti-*T. gondii* IgG antibodies and 36 (20.8%) were also positive for anti-*T. gondii* IgM antibodies. Of the 50 anti-*T. gondii* IgG positive participants, 31 (62%) had IgG levels higher than 150 IU/ml, 2 (4%) between 100 to 150 IU/ml, and 17 (34%) between 9 to 99 IU/ml. General socio-demographic characteristics of the 173 migrant workers and their relation with *T. gondii* seropositivity are shown in Table 1. All workers had a low socioeconomic status. Most participants were born in Durango, Mexico. The mean age of participants was 34.82 ± 14.01 years (range 14–89 years). Seroprevalence of *T. gondii* exposure was not significantly influenced by age, gender, birth place, or educational level. In contrast, the seroprevalence of *T. gondii* infection was significantly higher in migrant workers residing in rural areas than those in urban or suburban areas (*P* = 0.04).

**Table 1:** **Socio-demographic characteristics of migrant agricultural workers and seroprevalence of *T. gondii *infection**

**Characteristic**	**No. of subjects**	**Prevalence of *T. gondii *infection**		***P***
	**tested**^**a**^	**No.**	**%**	**value**
Gender				
Male	95	29	30.5	0.6
Female	78	21	26.9	
Age groups (years)				
30 or less	79	17	21.5	0.14
31-50	68	24	35.3	
>50	26	9	34.6	
Birth place				
Durango State	160	47	29.4	0.75
Other Mexican State	13	3	23.1	
Residence place				
Durango State	169	50	29.6	0.32
Other Mexican State	4	0	0.0	
Residence area				
Urban or suburban	25	3	12.0	0.04
Rural	147	47	32.0	
Educational level				
No education	35	7	20.0	0.53
1-6 years	81	25	30.9	
7-12 years	56	18	32.1	
13 or more years	1	0	0.0	

^a^Sums may not add up to 173 because of a missing value.

With respect to clinical data (Table 2), migrant workers suffering from memory impairment had significantly higher seroprevalences of anti-*T. gondii* IgG than those without this clinical feature (*P* = 0.007). In addition, migrant workers suffering from dizziness or syncope had significantly higher seroprevalence of anti-*T. gondii* IgG antibodies than those without such clinical characteristics (*P* = 0.02). The frequencies of other clinical characteristics including presence or history of lymphadenopathy, reflex, hearing and visual impairments, surgery history, blood transfusion, and transplant history were similar among *T. gondii* positive and *T. gondii* negative individuals. Abortion history in women was not associated with *T. gondii* seropositivity. With respect to IgM seroprevalence, further analysis of clinical data showed that workers suffering from memory impairment also had a significantly (*P* = 0.02) higher seroprevalence of anti-*T. gondii* IgM antibodies (16/51: 31.4%) than those without such clinical features (20/122: 16.4%). Other clinical characteristics were not associated with *T. gondii* IgM seropositivity.

**Table 2:** **Bivariate analysis of clinical data and infection with *T. gondii *in migrant agricultural workers of Durango State, Mexico**

**Characteristic**	**No. of subjects**	**Prevalence of *T. gondii *infection**		***P***
	**tested**^**a**^	**No.**	**%**	**value**
Clinical status				
Healthy	140	39	27.9	0.53
Ill	33	11	33.3	
Lymphadenopathy ever				
Yes	45	14	31.1	0.70
No	128	36	28.2	
Headache frequently				
Yes	74	27	36.5	0.05
No	99	23	23.2	
Dizziness or syncope				
Yes	5	4	80	0.02
No	168	46	27.4	
Memory impairment				
Yes	51	22	43.1	0.007
No	122	28	23	
Reflex impairment				
Yes	36	13	36.1	0.28
No	137	37	27	
Hearing impairment				
Yes	19	5	26.3	0.79
No	154	45	29.2	
Visual impairment				
Yes	48	18	37.5	0.12
No	125	32	25.6	
Surgery ever				
Yes	40	10	25	0.51
No	132	40	30.3	
Blood transfusion				
Yes	23	8	34.8	0.50
No	150	42	28	
Transplantation				
Yes	2	1	50	0.49
No	171	49	28.7	
Abortion in women				
Yes	19	5	26.3	0.58
No	55	14	25.5	

^a^Sums may not add up to 173 because of a missing value.

Concerning behavioral characteristics (Table 3), the bivariate analysis showed a number of variables with a *P* value equal to or less than 0.20 including traveling abroad (*P* = 0.01), national trips (*P* = 0.003), consumption of pork (*P* = 0.03), goat meat (*P* = 0.19), turkey meat (*P* = 0.14), opossum meat (*P* = 0.12), raw milk (*P* = 0.05), unwashed raw vegetables (*P* = 0.13), and a low frequency (up to 10 times a year) of eating out of home (*P* = 0.008). Other behavioral variables including animal contacts, consumption of raw or uncooked meat, and untreated water showed *P* values higher than 0.20 in the bivariate analysis. Further analysis by using logistic regression (Table 4) showed that *T. gondii* exposure was positively associated with consumption of unwashed raw vegetables (OR = 2.39; 95% CI: 1.06-5.35; *P* = 0.03) and low frequency of eating out of home (OR = 3.87; 95% CI: 1.43-10.42; *P* = 0.007), and was negatively associated with national trips (OR = 0.30; 95% CI: 0.13-0.65; *P* = 0.003) and consumption of raw milk (OR = 0.40; 95% CI: 0.18-0.87; *P* = 0.02). Consumption of opossum meat was not included in the multivariate analysis because none of the 6 consumers were positive for anti-*T. gondii* antibodies.

**Table 3:** **Bivariate analysis of selected putative risk factors for infection with *T. gondii *in migrant agricultural workers in Durango, Mexico**

**Characteristic**	**No. of subjects**	**Prevalence of *T. gondii *infection**		***P***
	**tested **^**a**^	**No.**	**%**	**value**
Cats at home				
Yes	87	22	25.3	0.29
No	86	28	32.6	
Cleaning cat excrement				
Yes	68	19	27.9	0.81
No	98	29	29.6	
Traveled abroad				
Yes	17	1	5.9	0.01
No	156	49	31.4	
National trips				
Yes	98	20	20.4	0.003
No	73	30	41.1	
Pork consumption				
Yes	158	49	31	0.03
No	15	1	6.7	
Goat meat consumption				
Yes	87	29	33.3	0.19
No	86	21	24.4	
Mutton consumption				
Yes	49	11	22.4	0.23
No	124	39	31.5	
Turkey meat consumption				
Yes	70	16	22.9	0.14
No	103	34	33	
Duck meat consumption				
Yes	24	9	37.6	0.31
No	149	41	27.5	
Venison consumption				
Yes	132	40	30.3	0.46
No	41	10	24.4	
Opossum meat consumption				
Yes	6	0	0	0.12
No	167	50	29.9	
Frequency of meat consumption				
Never	2	0	0	0.66
Up to 3 times a week	161	47	29.2	
4-7 times a week	10	3	30	
Degree of meat cooking				
Raw	1	0	0	0.46
Undercooked	8	1	12.5	
Well done	164	49	29.9	
Raw milk consumption				
Yes	86	19	22.1	0.05
No	87	31	35.6	
Sausages consumption				
Yes	145	44	30.3	0.34
No	28	6	21.4	
Salami consumption				
Yes	37	9	24.3	0.48
No	136	41	30.1	
Unwashed raw vegetables				
Yes	61	22	36.1	0.13
No	111	28	25.2	
Unwashed raw fruits				
Yes	73	22	30.1	0.71
No	98	27	27.6	
Untreated water				
Yes	108	34	31.5	0.34
No	61	15	24.6	
Frequency of eating out of home				
Up to 10 times a year	123	43	35	0.008
>10 times a year	48	7	14.6	
Soil contact				
Yes	165	49	29.7	0.27
No	8	1	12.5	
Floor at home				
Ceramic or Wood	28	7	25	0.45
Concrete	98	32	32.7	
Soil	47	11	23.4	

^a^Sums may not add up to 173 because of some missing values.

**Table 4:** **Multivariate analysis of selected characteristics of migrant agricultural workers and their association with *T. gondii *infection**

**Characteristic**	**Odds ratio**	**95% confidence interval**	***P***
			**value**
Travel abroad	0.17	0.02-1.51	0.11
National trips	0.30	0.13-0.65	0.003
Consumption of:			
Pork	6.25	0.65-60.01	0.11
Goat meat	1.83	0.82-4.05	0.13
Turkey meat	0.47	0.21-1.06	0.07
Raw milk	0.40	0.18-0.87	0.02
Unwashed raw vegetables	2.39	1.06-5.35	0.03
Low frequency of eating out of home	3.87	1.43-10.42	0.007

## Discussion

The 28.9% seroprevalence of *T. gondii* exposure found in migrant agricultural workers is comparable with those (22.4%-30.3%) found in other rural communities in Durango, Mexico, including the general population in 3 rural communities [8], Mennonites [9] and Tepehuanos [13]. However, the seroprevalence found in migrant workers is higher than seroprevalences reported in urban populations in Durango City. The seroprevalence of *T. gondii* infection ranged between 6.1% and 12% among pregnant women [14], healthy blood donors [15], subjects suffering from a number of underlying diseases [16], and the general population in Durango City [10]. It is likely that differences in sanitation between urban and rural communities may contribute to the differences in seroprevalences. All migrant agricultural workers live in poverty and have poor housing conditions including soil floors at home and lack of urban services including potable water and drainage. In addition, when they work in seasonal agricultural labor out of their homes they live in shelters with very low hygiene and sanitary conditions too. To our knowledge there are no previous study on internal migrant agricultural workers in other Mexican states or other countries, therefore, we cannot compare our results with those of other studies. In a regional context, the seroprevalence of *T. gondii* infection found in migrant agricultural workers in Durango is higher than those (20.3%-23.5%) found in neighboring Mexican states (Chihuahua, Coahuila and Zacatecas) in a national seroepidemiology survey [17]. However, comparison of these seroprevalences should be interpreted with care since different laboratory methods among the studies were used. We used an enzyme immunoassay while an indirect immunofluorescence test was used in the national survey. In a national context, the seroprevalence of *T. gondii* infection in migrant agricultural workers in Durango is comparable with the mean national seroprevalence (27.97%) of *T. gondii* infection reported in Mexico [18].

Of the behavioral characteristics, *T. gondii* exposure was positively associated with consumption of unwashed raw vegetables. This finding indicates that parasite transmission might have occurred by ingesting *T. gondii* oocysts. Shelters for migrant workers had cats. However, it is not clear whether infection occurred during their stay in the shelters or elsewhere. Transmission by parasite cysts was less likely to occur; migrant workers do not eat meat frequently because they cannot afford to buy it. The very high seroprevalence of anti-*T. gondii* IgM antibodies found in migrant workers suggests recent infections. Nevertheless, interpretation of the increased IgM seroprevalence should be taken with care since tests for detection of IgM antibodies may have a high rate of false positive results caused by limitations in test specificity or persistence of IgM antibodies [19]. Some known clinical features of toxoplasmosis including visual impairment and headaches [2,20-22] were observed in some migrant workers. In fact, frequent headaches showed a borderline (*P* = 0.05) association with *T. gondii* seropositivity. Of note, seropositivity to both anti-*T. gondii* IgM and IgG antibodies were associated with memory impairment. This association has been previously reported in gardeners [23]. In addition, we found an association of seropositivity to anti-*T. gondii* IgG antibodies with dizziness or syncope. Altogether, results indicate that both recent and latent toxoplasmosis cases were present among migrant agricultural workers. The impact of *T. gondii* on the health of migrant workers may have important labor implications. Migrant workers are paid for piecework; therefore, workers suffering from symptomatic toxoplasmosis may be affected by a reduction in their performance and income. Furthermore, *T. gondii* infection in workers is of concern since such infection has been associated with reflex impairment [24] and work accidents [25].

The positive associations of a low frequency of eating out of home and consumption of unwashed raw vegetables, and the negative association with national trips suggest that infection was most likely acquired at home in Durango by eating contaminated vegetables. Therefore, health care providers should focus their attention on educational strategies aimed to improve food hygiene practices in migrant agricultural workers. The negative association of *T. gondii* seropositivity with consumption of raw milk suggests that this behavioral characteristic did not play an important role in the transmission of *T. gondii* infection in migrant agricultural workers.

A limitation of the study was that only one Mexican state was explored. However, results of this first study represent the baseline information on the seroprevalence and contributing factors for *T. gondii* infection in internal migrant agricultural workers in Mexico and other countries. Results of the present work are of concern since migrant agricultural workers play an important role in the economy of many countries. Results warrant for further research.

## Conclusions

We conclude: 1) the seroprevalence of *T. gondii* exposure in migrant agricultural workers living in poverty is comparable to that reported in other rural populations in the region; however, seroprevalence is higher than that reported in the nearby urban capital city; 2) *Toxoplasma* may impact on the health of migrant workers. Our results may have labor implications and will help to design optimal preventive measures against *T. gondii* infection.

## Competing interests

The authors declare that they have no competing interests.

## Authors’ contributions

CAE conceived and designed the study protocol, participated in the coordination and management of the study, applied the questionnaires, performed the laboratory tests and data analysis, and wrote the manuscript. FCR obtained clinical data, applied the questionnaires and performed the data analysis. OL performed the data analysis, and wrote the manuscript. All authors read and approved the final version of the manuscript.
